# The Association between Progression of Atherosclerosis and the Methylated Amino Acids Asymmetric Dimethylarginine and Trimethyllysine

**DOI:** 10.1371/journal.pone.0064774

**Published:** 2013-05-29

**Authors:** Kjetil H. Løland, Øyvind Bleie, Heidi Borgeraas, Elin Strand, Per M. Ueland, Asbjørn Svardal, Jan E. Nordrehaug, Ottar Nygård

**Affiliations:** 1 Department of Clinical Science, University of Bergen, Bergen, Norway; 2 Department of Heart Disease, Haukeland University Hospital, Bergen, Norway; 3 Morbid Obesity Centre, Vestfold Hospital Trust, Tønsberg, Norway; College of Pharmacy, University of Florida, United States of America

## Abstract

**Objective:**

We previously showed that treatment with folic acid (FA)/B_12_ was associated with more rapid progression of coronary artery disease (CAD). High doses of FA may induce methylation by increasing the availability of S-adenosyl-methionine (SAM). Asymmetric dimethylarginine (ADMA) and trimethyllysine (TML) are both produced through proteolytic release following post-translational SAM–dependent methylation of precursor amino acid. ADMA has previously been associated with CAD. We investigated if plasma levels of ADMA and TML were associated with progression of CAD as measured by quantitative coronary angiography (QCA).

**Methods:**

183 patients from the Western Norway B Vitamin Intervention Trial (WENBIT) undergoing percutaneous coronary intervention (PCI) were randomized to daily treatment with 0.8 mg FA/0.4 mg B_12_ with and without 40 mg B_6_, B_6_ alone or placebo. Coronary angiograms and plasma samples of ADMA and TML were obtained at both baseline and follow-up (median 10.5 months). The primary end-point was progression of CAD as measured by diameter stenosis (DS) evaluated by linear quantile mixed models.

**Results:**

A total of 309 coronary lesions not treated with PCI were identified. At follow-up median (95% CI) DS increased by 18.35 (5.22–31.49) percentage points per µmol/L ADMA increase (p-value 0.006) and 2.47 (0.37–4.58) percentage points per µmol/L TML increase (p-value 0.021) in multivariate modeling. Treatment with FA/B_12_ (±B_6_) was not associated with ADMA or TML levels.

**Conclusion:**

In patients with established CAD, baseline ADMA and TML was associated with angiographic progression of CAD. However, neither ADMA nor TML levels were altered by treatment with FA/B_12_ (±B_6_).

**Trial Registration:**

Controlled-Trials.com NCT00354081

## Introduction

Hyperhomocysteinemia is a significant risk factor for coronary artery disease (CAD) in epidemiological studies. However, several large-scale clinical trials with homocysteine-lowering B-vitamins have repeatedly demonstrated no clinical benefit of the intervention. [1]–[3] On the contrary, pooled analyses suggest increased cardiovascular mortality associated with B-vitamin treatment in certain sub-groups [2] as well as increased cancer incidence and all-cause mortality. [4] We have previously shown that a sub-group of patients with established CAD had a more rapid progression of sub-clinical atherosclerosis as measured by quantitative coronary angiography (QCA), when receiving 0.8 mg folic acid (FA) and 0.4 mg vitamin B_12_ daily. [5] FA and vitamin B_12_ supplementation promotes remethylation of homocysteine to methionine and subsequently increases S-adenosyl-methionine (SAM), which is the main methyl donor in cellular transmethylation reactions. [6].

A growing body of evidence has shown that both global and site-specific hypo- and hypermethylation of DNA and histones are associated with cardiovascular disease (CVD). [6] High doses of FA induce aberrant DNA methylation in some [7] but not all studies. [8] It has been speculated that the lack of cardiovascular protective effect of homocysteine-lowering FA supplementation is due to a simultaneously increased methylation potential and subsequent epigenetic alterations of gene expression by folate. [9] Histone methylation is limited to the ε-amino groups of amino acid residues in the form of mono-, di- or trimethylation, [6] and only two amino acids in histones undergo methylation, i.e. arginine and lysine. [10].

Asymmetric dimethylarginine (ADMA), a product of proteolytic degradation of methylated proteins such as histones, is a well-known inhibitor of nitric oxide synthase (NOS) and has been associated with endothelial dysfunction and CVD. [11]–[14] ADMA levels have not, to our knowledge, been linked to global methylation status. 6-N-trimethyllysine (TML), a precursor in carnitine synthesis, [15], [16] is another methylated amino acid which is produced through the lysosomal or proteasomal degradation of proteins such as histones containing methylated amino acids residues, specifically trimethylated lysine. [16].

We investigated whether ADMA and TML could serve as predictive markers of progression of CAD as measured by QCA and if treatment with FA-vitamin B_12_ is related to ADMA or TML levels.

## Methods

### Study Design and Patient Population

The current study included patients who participated in the Western Norway B Vitamin Intervention Trial (WENBIT). WENBIT was a double-blinded, placebo-controlled, two-centre trial conducted among 3090 adult patients (20.5% women) having undergone coronary angiography for suspected CAD. Details, inclusion and exclusion criteria, and the main results of the trial have been described previously [1] In order to simultaneously evaluate the effect of FA/vitamin B_12_ and vitamin B_6_, patients were randomly assigned into 4 groups, using 2 by 2 factorial design, to daily receiving an oral capsule with one of the following compositions: 1) FA 0.8 mg, vitamin B_12_ (cyanocobalamin) 0.4 mg and vitamin B_6_ (pyridoxine) 40 mg, or 2) FA plus vitamin B_12_, or 3) vitamin B_6_, or 4) placebo. In this study we evaluated the effect of FA/vitamin B_12_ on plasma levels of ADMA and TML. Intervention groups 1) and 2) were thus compared to 3) and 4) in the subsequent analyses.

In the current sub-study, patients at high risk of procedural complications, or those presenting with a baseline coronary anatomy of such a nature that re-angiography probably would prove unsuccessful, were also excluded. Further exclusion criteria were baseline and/or follow-up coronary angiograms considered unsuitable for quantitative analysis.

At Haukeland University Hospital a total of 570 patients underwent percutaneous coronary intervention (PCI) following the baseline angiography during the sub-study inclusion time period and were eligible for re-angiography, of which 371 (65%) underwent re-angiography as previously described. [5] A total of 23 (6%) participants were excluded from the analysis due to technically inadequate angiograms and 165 (47%) participants did not fulfill QCA lesion criteria. We included 183 patients with adequate serial QCA data who had at least one qualifying lesion, resulting in a total of 309 coronary lesions for analyses.

### Ethics Statement

All clinical investigation was conducted according to the principles expressed in the Declaration of Helsinki. Written informed consent was obtained from all WENBIT participants, and an additional written informed consent was collected from patients scheduled for re-angiography. Both WENBIT and the current sub-study were approved by the Regional Committee for Medical and Health Research Ethics (Regional Ethics Committee [REC] West/Regional Etisk Komité [REK] vest, which is the institutional review board available at http://helseforskning.etikkom.no), the Norwegian Medicines Agency (Legemiddelverket) and by the Data Inspectorate (Datatilsynet). WENBIT was registered with ClinicalTrials.gov with identifier NCT00354081 and URL http://www.clinicaltrials.gov/ct2/show/NCT00354081. WENBIT was a two-centre study conducted at Haukeland University Hospital, Bergen, and Stavanger University Hospital, Stavanger, both located in western Norway. All clinical research was conducted in Norway and was subject to Norwegian law.

### Quantitative Coronary Angiography

Both baseline and follow-up coronary angiograms were analysed using QCA by two trained technicians blinded to the treatment regimen and supervised by an experienced interventional cardiologist. A total of 16 coronary artery segments were evaluated in all patients; i.e. 15 segments as per the American Heart Association standardization criteria, [17] plus the right atrioventricular branch. Eligible lesions for analysis had a reference diameter ≥2 mm, diameter reduction of ≥30% at either baseline or follow-up, and were adequately visualized at similar projections at both procedures. The analysed segment had not been treated with PCI. Cases of disagreement between the observers, about eligibility of a certain lesion, were subject to reanalyses by both observers. Following all QCA procedures, segments from both observers were compared to ensure equality concerning accurate numbering of segments, the correct angiogram analysed, and the actual stenosis portrayed.

Lesions were analysed using digitalized QCA (Quantcor QCA [CAAS II V 5.0], Pie Medical Imaging, the Netherlands). An end-diastolic frame showing the stenosis without foreshortening or vessel overlap, and free of intra-coronary wires, was selected. If the stenosis differed in severity in different projections, the projection demonstrating the most severe stenosis was subject to analysis. The contrast-filled tip of the catheter was used for calibration, and computer-defined obstruction analysis without manual contour correction was used where applicable. However, ostial stenoses required the use of manually-defined obstruction analysis (user-defined reference vessel diameter and stenosis length), while branched artery segment required manual correction of vessel contour.

### Follow-up and End-point

The primary measure for each selected lesion was diameter stenosis (DS) [18], [19] in percentages, measured as continuous variables and defined as the mean of the values measured separately by two different observers.

When all baseline and follow-up lesions had been analysed by both observers, the inter-observer difference in DS was calculated. The 10% of lesions with the largest difference were subject to re-analysis.

The primary angiographic end-point of the study was defined as DS measured at follow-up, while baseline values were used for adjustment. I.e. change in DS was the *de facto* end-point.

### Blood Samples

Blood samples were collected at baseline and at the re-angiography. Routine blood analyses such as hematologic parameters, renal function markers and lipid-related factors were analysed in fresh samples at the Laboratory of Clinical Biochemistry, Haukeland University Hospital, by standard methods, all blinded to study end-points and randomization. Blood samples for measurements of ADMA, B vitamins and associated compounds were analysed at the laboratory of Bevital AS (www.bevital.no), Bergen, Norway using methods previously described. [20] Plasma TML, free carnitine and γ-butyrobetaine were analysed using MS/MS as described previously [21] with some modifications of the high-performance liquid chromatography (HPLC) conditions. Estimation of glomerular filtration (eGFR) rate was done using the simplified MDRD-equation. [22].

### Statistical Analysis

Analyses were conducted according to the intention-to-treat principle. Continuous variables are reported as means ± SD or as median (interquartile range) where appropriate. Categorical variables are presented as numbers (percentages).

Differences between intervention groups in continuous variables were analysed using Student’s *t*-test or Mann Whitney U-test. Differences in categorical variables were analysed by chi-square test or Fisher’s exact test. Inter-observer reliability were assessed on QCA measurements by calculating the average measure intra-class coefficient [23]. The intra-class correlation coefficient (ICC) of ADMA and TML was calculated using samples one year apart and subjects were considered as random effects (“oneway” model). Degree of correlation between covariates was assessed using Spearman’s rank correlation coefficient.

In order to assess for non-linear relationships between outcome and explanatory variables, we first fitted a generalized additive mixed model (data not shown). A linear relationship provided the best model fit, thus we proceeded to use a simpler linear model.

As several lesions stemmed from the same patients – i.e. 309 lesions from 183 patients, our data was not independent. We have previously addressed this problem using a mixed effects model allowing for random effects. [5].

Because of non-normal distributions we used a non-parametric test, i.e. conditional quantile regression (CQR). CQR is the estimation of an unknown quantile of an outcome as a function of a set of covariates where the response is assumed to follow an asymmetrical Laplace distribution and using a likelihood-based approach. [24] Bootstrapping is used to estimate the variance of the covariates.

We estimated the median DS as a function of fixed and random effects using a linear quantile mixed model (LQMM). A total of three models were fitted, i.e. two bivariate models with baseline measurement of ADMA or TML and DS as explanatory variables and a multivariate model which in addition included baseline measurements of age, gender, FA/B_12_ intervention status (yes/no), follow-up time (days), diabetes (yes/no), current smoking (yes/no), systolic blood pressure (mmHg), body mass index (kg/m^2^), eGFR (mL/min/1.73 m^2^), apolipoprotein B100 (g/L) and C-reactive protein (mg/L). Estimation of the 10^th^, 25^th^, 50^th^ (median), 75^th^ and 90^th^ percentile was performed for all models.

Using a similar LQMM, we assessed the effect of FA/vitamin B_12_ supplementation on measurements of ADMA or TML at follow-up as outcome variable and ADMA or TML at baseline and FA/vitamin B_12_ randomization status as explanatory variables. The clustering of coronary segments within lesions was the random effect.

LQMM was applied using the *lqmm* package [25] in *R* version 2.15.0 (R Development Core Team; Vienna, Austria), implemented as described by Geraci and Bottai. [24] Intra-class correlation coefficients were calculated using the R package, *irr* version 0.84. For all analyses, a p-value <0.05 was considered statistically significant.

## Results

### Baseline Characteristics

Baseline demographic, clinical and laboratory characteristics (Table 1) showed higher systolic blood pressure, more extracardial disease as well as less statin and betablocker use in the B6/placebo group. Ninety-eight percent of the patients were treated with statins. Median (interquartile range [IQR]) age was 60.0 (14.0) years, 15.8% were women and 27.3% of the patients had a history of prior myocardial infarction. Median (IQR) serum total cholesterol was 5.0 (1.3) mmol/L, serum triglycerides 1.54 (0.87) mmol/L and serum CRP 2.0 (4.9) g/L. The median (IQR) concentrations were 0.51 (0.11) µmol/L for ADMA, 0.86 (0.36) µmol/L for TML and 10.3 (6.0) nmol/L for folate.

**Table 1:** Baseline Characteristics and Laboratory Findings in Patients with Angiographic Coronary Lesions (n = 183).

Characteristics		Group 1 (n = 98)FA, B_12_±B_6_	Group 2 (n = 85)Placebo or B_6_	P-value
**Demographic characteristics**				
	Age - years	59.3 (10.5)	61.5 (9.4)	0.14
	Female sex - no. (%)	17 (17.3)	12 (14.1)	0.69
**Clinical characteristics**				
	Systolic Blood Pressure - mmHg	138.3 (20.7)	147.4 (23.9)	0.01
	Body Mass Index - m^2^/kg	27.0 (3.2)	27.2 (3.5)	0.79
	Ejection Fraction - %^a^	60.7 (7.4)	61.6 (9.2)	0.45
**Cardiovascular risk factors - no. (%)**				
	Stable angina at baseline angiography	67 (68.4)	59 (69.4)	0.88
	NSTACS at presentation	31 (31.6)	26 (30.6)	0.88
	Extracardial vascular disease^b^	7 (7.1)	15 (17.6)	0.03
	Prior AMI	24 (24.5)	26 (30.6)	0.36
	Prior PCI	22 (22.4)	12 (14.1)	0.15
	Prior CABG	5 (5.1)	1 (1.2)	0.14
	Hypercholesterolaemia	58 (59.2)	58 (68.2)	0.20
	Hypertension	40 (40.8)	40 (57.6)	0.40
	Diabetes Mellitus (type I and II)	7 (7.1)	10 (11.8)	0.28
	Current smoker	34 (34.7)	27 (31.8)	0.68
	Disease severity			0.90
	* 1-vessel disease*	36 (36.7)	31 (36.5)	1.00
	* 2-vessel disease*	41 (41.8)	38 (44.7)	0.77
	* 3-vessel disease*	21 (21.4)	16 (18.8)	0.71
**Medical therapy** c **- no. (%)**				
	Statins	98 (100.0)	81 (95.2)	0.03
	β-adrenergic receptor antagonists	82 (83.7)	57 (67.0)	0.01
	Calcium antagonists	18 (18.4)	11 (12.9)	0.32
	ACE-inhibitors^d^	12 (12.2)	19 (22.4)	0.07
	Acetylsalisylic acid	96 (98.0)	85 (100.0)	0.19
	ADP receptor antagonists	92 (93.9)	79 (92.9)	0.80
**Laboratory findings**				
	S-C-Reactive Protein - mg/L	1.8 (4.9)	2.5 (4.9)	0.40
	S-LDL cholesterol - mmol/L	3.0 (1.3)	3.1 (1.1)	0.39
	S-HDL cholesterol - mmol/L	1.2 (0.4)	1.2 (0.4)	0.76
	Apolipoprotein B100 - g/L	0.87 (0.34)	0.88 (0.28)	0.33
	eGFR – mL/min/1.73 m^2^	95 (17)	93 (19)	0.34
	Serum glucose - mmol/L	5.5 (1.3)	5.7 (1.7)	0.72
	Plasma -homocysteine - µmol/L	9.9 (3.1)	9.8 (3.3)	0.27
	Plasma folate - nmol/L	10.1 (5.8)	11.0 (6.3)	0.20
	Asymmetric dimethylarginine - µmol/L	0.50 (0.09	0.52 (0.12)	0.23
	S-Trimethyllysine - µmol/L	0.85 (0.34)	0.87 (0.40)	0.48
	Carnitine – µmol/L	40.5 (7.5)	41.0 (10.1)	0.52
	γ-Butyrobetaine – µmol/L	0.98 (0.30)	1.02 (0.28)	0.05

For continuous variables, mean and standard deviation or median and interquartile range within each group is calculated. Student's T-test or Mann-Withney U-test was used to compare the two groups. For categorical variables, number and percentage is presentend and a Chi square test was used to compare the four groups. Fisher's exact test was used when appropriate. All biochemical parameteres are prestented as median (interquartile range). FA, folic acid (0.8 mg); B_12_, vitamin B_12_ (0.4 mg); B_6_, vitamin B_6_ (40 mg); PCI, percutaneous coronary intervention; CABG, coronary artery bypass graft surgery; NSTACS, composite syndrom consisting of acute coronary syndrome including both ST-elevated and non-ST-elevated myocardial infarction; AMI, acute myocardial infarction; CHD, coronary heart disease; LMS, left main stem; LAD, left anterior descending artery; CX, circumflex branch; RCA, right coronary artery; ACE, Angiotensin I converting enzyme; LDL, low-density lipoprotein; HDL, high-density lipoprotein; eGFR, estimated glomerular filtration rate; ADMA, asymmetric dimethylarginine. Percentages may not add up due to rounding of numbers.

a Ejection fraction was measured during ventriculography for the majority of the patients. When this was not performed, ultrasonic echocardiography was used.

b A prior diagnosis of any peripheral or cerebrovascular disease.

c Medication at discharge.

d Including ARB - angiotensin receptor blockers.

### Angiographic Findings

A total of 309 coronary lesions from 183 patients were finally identified by both observers to comply with the criteria for DS analysis, and angiographic characteristics are shown in table 2. We observed progression of CAD during follow-up as indicated by a statistically significant change in DS. Among 309 lesions median (95% CI) DS increased by 3.51 (2.26–4.76) percentage points (p<0.0001).

**Table 2:** Baseline Angiographic Characteristics of the 309 Coronary Lesions (from n = 183 patients).

Characteristic		Group 1 (n = 152)FA, B_12_±B_6_	Group 2 (n = 157)Placebo or B_6_	P Value
**Selection - no. (%)**				
	Included coronary lesions	152 (49.2)	157 (50.8)	−
	Patients	98 (53.6)	85 (46.4)	−
	LMS lesions	2 (1.3)	1 (0.6)	0.62
	LAD lesions	38 (25.0)	31 (19.7)	0.33
	CX lesions	34 (22.4)	48 (30.6)	0.13
	RCA lesions	78 (51.3)	77 (49.0)	0.78
**Analysed segment morphology**				
	Length analysed segment - mm	21.0 (7.2)	21.4 (6.5)	0.58
	Length stenotic segment - mm	10.2 (4.2)	10.1 (4.3)	0.80
	Reference diamenter - mm	3.09 (0.82)	3.08 (0.69)	0.89
	Minimum lumen area - mm^2^	4.16 (2.76)	3.95 (2.26)	0.45
	Volume stenosis - mm^3^	29.3 (11.9)	29.9 (11.9)	0.66
	Minimum lumen diameter - mm	1.94 (0.61)	1.90 (0.49)	0.51
	Diamenter stenosis - %	37.2 (9.7)	38.0 (9.6)	0.47

For continuous variables, mean and standard deviation within each group is calculated. Student's T-test was used to compare the two groups. For categorical variables, number and percentage is presentend and a Chi square test used to compare the two groups. Fisher's exact test was used when appropriate. ). FA, folic acid (0.8 mg); B_12_, vitamin B_12_ (0.4 mg); B_6_, vitamin B_6_ (40 mg); LMS, left main stem artery; LAD, left anterior descending artery; CX; circumflex artery; RCA, right coronary artery; mm, millimeters.

### Angiographic Progression According to Plasma Asymmetric Dimethylarginine and Trimethyllysine

**Figure 1:**
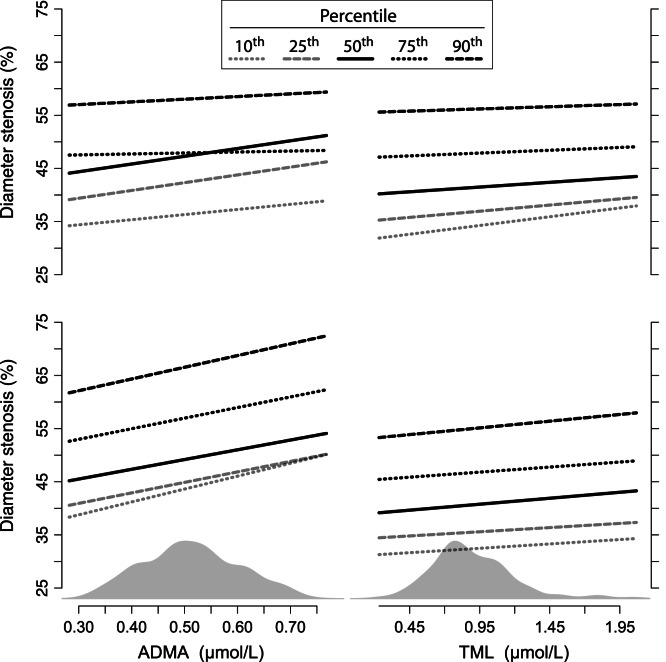
Asymmetric dimethylarginine, trimethyllysine and angiographic progression of coronary artery disease. The graph shows the regression line from a linear quantile mixed model. The two left panels show the relation between the asymmetric dimethylarginine (ADMA) and DS measured at follow-up, whereas the two right panels show the relation between trimethyllysine (TML) and DS at follow-up. The bivariate models (adjusted for baseline DS measurement) are on the top and the multivariate (adjusted for age, sex, folic acid/B_12_ intervention status, follow-up time, diabetes, smoking, systolic blood pressure, body mass index, estimated glomerular filtration rate (eGFR), apolipoprotein B100, C-reactive protein, ADMA or TML) at the bottom. The plasma level of either ADMA or TML is shown on the x-axis, with DS at follow-up on the y-axis. The solid line represents the regression line for the effect on median DS, while the others are displayed according to the legend.

The main results are shown in table 3 as well as figure 1. Plasma levels of ADMA at baseline were, in the bivariate model, significantly related to DS at follow-up. Median (95% CI) DS increased by 14.57 (1.79–27.35) percentage points per µmol/L increase of ADMA (p-value 0.026). A similar statistically significant effect was found in the 25^th^ percentile of DS, while in the 10^th^, 75^th^ and 90^th^ percentile of DS there was non-significant relation to ADMA.

**Table 3:** Diameter stenosis at follow-up according to plasma concentrations of asymmetric dimethylarginine and trimethyllysine^a^.

Independentvariable	Effect estimates for each variable according to modeled percentile of diameter stenosis at follow-up^b^										
	10^th^ (31.5% DS)		25^th^ (35.0% DS)		Median [50^th^](40.0% DS)		75^th^ (47.0% DS)		90^th^ (55.5% DS)		
*Bivariate ADMA model* c											
ADMA at baseline -µmol/L	9.63 (−5.92–25.19)	0.22	14.63 (0.94–28.32)	0.04	14.57 (1.79–17.35)	0.03	1.81 (−15.77–19.40)	0.84	5.04 (−22.12–32.19)	0.71	
*Bivariate TML model* d											
TML at baseline -µmol/L	3.11 (0.64–5.58)	0.01	2.19 (−0.22–4.60)	0.07	1.68 (−0.15–3.52)	0.07	1.00 (−2.35–4.34)	0.56	0.78 (−4.32–5.89)	0.76	
*Multivariate mode* e											
ADMA – µmol/L	24.25 (7.40–41.10)	<0.01	19.75 (5.76–33.74)	<0.01	18.35 (5.22–31.49)	<0.01	19.93 (5.14–34.73)	<0.01	22.07 (6.50–37.65)	<0.01	
TML – µmol/L	1.81 (−0.99–4.61)	0.20	1.73 (−0.74–4.20)	0.17	2.47 (0.37–4.58)	0.02	2.09 (−0.69–4.87)	0.14	2.72 (−1.12–6.55)	0.16	

DS, Diameter Stenosis; ADMA, asymmetric dimethylarginine; TML, trimethyllysine.

a Non-parametric linear quantile mixed-effects models of diameter stenosis at follow-up using Laplace distribution.

b Effect estimate given as regression coefficient (95% confidence interval) and p-value for change in percentage point diameter stenosis.

c The fixed effect in this model is ADMA and DS measured at baseline while the random effect is the clustering of arterial segments within a single patient. Estimates are presented as median (95% confidence interval). Standard error is estimated using bootstrapping.

d The fixed effect in this model is TML and DS measured at baseline while the random effect is the clustering of arterial segments within a single patient. Estimates are presented as median (95% confidence interval). Standard error is estimated using bootstrapping.

e The fixed effect in this model is DS measured at baseline, follow-up time in days, presence of diabetes, randomization (folic acid/B_12_ vs no folic acid/B_12_) status at baseline, plasma TML at baseline, smoking status, age, gender, plasma ADMA at baseline, systolic blood pressure, body mass index, kidney function, apolioprotein B100 and C-reactive protein while the random effect is the clustering of arterial segments within a single patient. Standard error is estimated using bootstrapping.

Plasma TML at baseline was statistically significantly related to DS at follow-up in the bivariate model at the 10^th^ percentile of DS, which increased by 3.11 (0.64–5.58) percentage point per µmol/L increase of TML (p-value 0.01). At the 25^th^, median, 75^th^ and 90^th^ percentile of DS there was non-significant relation to TML.

In the multivariate model both ADMA and TML at baseline was independently associated with DS at follow-up (Table 3). The median DS increased by 18.35 (5.22–31.49) percentage points per µmol/L increase of ADMA (p-value 0.006) and 2.47 (0.37–4.58) percentage points per µmol/L increase of TML (p-value 0.021). ADMA was significantly related to the 10^th^, 25^th^, 75^th^ and 90^th^ percentile of DS, while TML was not. In addition, male gender, C-reactive protein and eGFR were significantly related to DS.

Inclusion of eGFR in the multivariate model increased both the estimates and level of significance for both ADMA and TML regarding estimation of DS. When adjusting for γ-butyrobetaine or carnitine in the multivariate model, no significant change to the ADMA or TML estimates or levels of significance occurred (data not shown). In separate analyses without inclusion of ADMA or TML, neither γ-butyrobetaine nor carnitine predicted DS in bi- or multivariate analysis.

At baseline, the mean (SD) length of the included lesions was 21.2 (6.8) mm with a reference diameter of 3.08 (0.75) mm, minimum lumen diameter 1.92 (0.55) mm while DS was 37.6 (9.6) %.

### Assessment of Correlation between Covariates

At baseline Spearman’s ranks correlation coefficient between ADMA and TML was 0.27 (p-value <0.0001). ADMA showed the following correlation coefficients with other covariates: γ-butyrobetaine 0.29 (p-value <0.0001), carnitine 0.08 (p-value 0.15), creatinine 0.17 (p-value 0.003) and eGFR −0.35 (p-value <0.0001). TML significantly correlated (all p-value <0.0001) with γ-butyrobetaine, carnitine, creatinine and eGFR with a correlation coefficient of 0.41, 0.22, 0.28 and −0.26 respectively.

In addition, γ-butyrobetaine correlated with carnitine 0.42 (p-value <0.0001), eGFR 0.42 (p-value <0.0001) and creatinine 0.54 (p-value <0.0001). Carnitine did not significantly correlate with eGFR (0.08; p-value 0.14) or creatinine (0.01; p-value 0.80).

### Asymmetric Dimethylarginine and Trimethyllysine According to B Vitamin Supplementation

**Figure 2:**
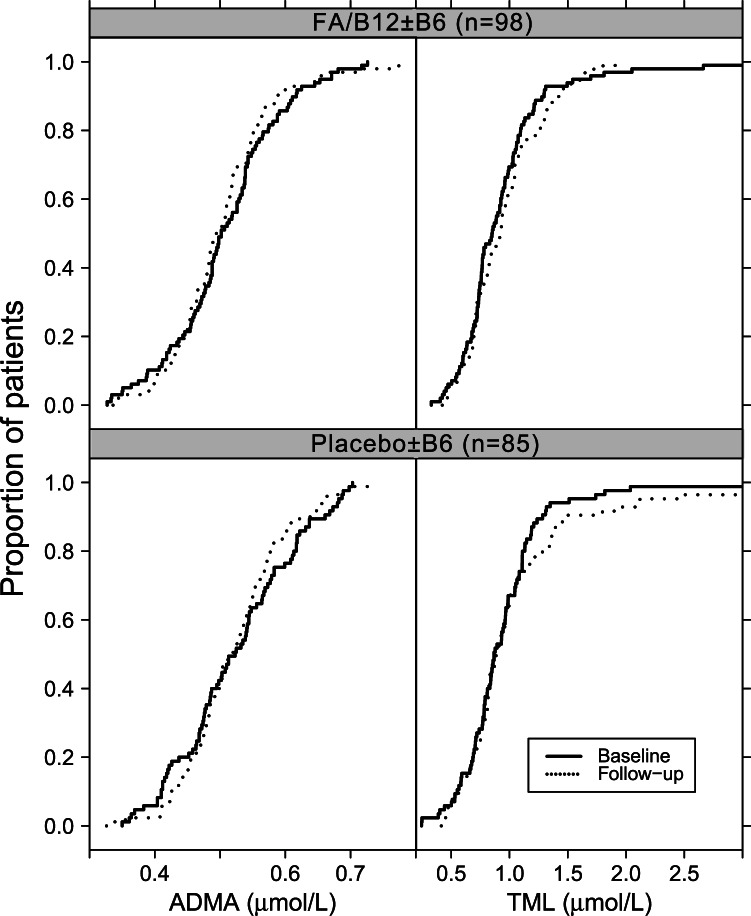
Asymmetric dimethylarginine and trimethyllysine before and after supplementation with folic acid/vitamin B_12_. The graph shows empirical cumulative distribution frequencies for asymmetric dimethylarginine on the left and trimethyllysine on the right. Patients receiving folic acid/B_12_ are displayed on the top, while patients receiving placebo or B_6_ on the bottom. Time of measurement is shown as baseline (solid line) and follow-up (dashed line) after a median of 10.5 month.

Cumulative distribution frequency plots showing the differences in ADMA and TML between baseline and follow-up during the B-vitamin treatment are shown in figure 2 for both intervention groups. By visual inspection there was no apparent difference between baseline and follow-up measurements of neither ADMA nor TML levels between the two treatment groups. In LQMM, FA/vitamin B_12_ treatment did not result in a statistically significant change in ADMA levels for the 25^th^, median, 75^th^ or 90^th^ percentile. The 10^th^ percentile of ADMA did however increase with 0.040 (0.002–0.078) µmol/L in patients receiving FA/vitamin B_12_ (p-value 0.04). No percentile (10^th^, 25^th^, median, 75^th^ or 90^th^) of TML was altered by FA/vitamin B_12_ treatment. Vitamin B_6_ treatment did not alter either ADMA or TML levels (data not shown).

### Intra-individual Stability of Biomarkers

Repeated measurements of ADMA and TML were available. One-way intra-class correlation coefficient (ICC) was 0.54 (95% CI 0.43–0.64) for ADMA and 0.37 (95% CI 0.24–0.49) for TML.

### Patient Follow-up

Patients were followed for median (IQR) 316 (78) days. At sub-study end, patients assigned to FA/vitamin B_12_ had lowered homocysteine levels by 24.8% whereas patients receiving vitamin B_6_ alone or placebo had unchanged homocysteine levels compared to baseline).

## Discussion

In the current substudy of a large randomized clinical trial with moderate doses of oral FA and vitamin B_12_ we followed 183 patients treated with PCI for angiographic progression of CAD in non-treated lesions for a median of 10.5 months. A total of 309 coronary artery lesions as well as plasma levels of ADMA and TML were evaluated at both baseline and follow-up. Baseline levels of ADMA and TML was however independently associated with angiographic progression of CAD as measured by DS at follow-up in multivariate statistical models, with ADMA showing the strongest relationship. FA/vitamin B_12_ supplementation did not alter median ADMA or TML at follow-up.

In accordance with previous studies we found ADMA to be associated with increased risk of CVD, [11]–[14] an effect mainly thought to be mediated through inhibition of NOS and subsequent endothelial dysfunction. [11] ADMA was independently associated with angiographic progression of CAD over a wide distribution of DS using a multivariate model – i.e. the adverse effect of elevated ADMA was similar in respect to both the effect estimate and level of significance across the 10^th^, 25^th^, 50^th^, 75^th^ and 90^th^ percentile of DS. To our knowledge this association has not previously been shown for de novo atherosclerosis.

Like ADMA, TML is produced through post-translational methylation of amino acids in nuclear proteins [16] and subsequent release through proteolysis; yet associations of TML with CVD have not been addressed. Both previous speculations [9] and emerging evidence [6], [26], [27] suggest that epigenetic alterations of chromatin is relevant to the development and progression of atherosclerosis. Plasma levels of TML, in our material, were associated with angiographic progression of disease when we studied median DS in a multivariate model, but the effect was only borderline statistically significant in a bivariate model. In contrast to ADMA, TML levels is most likely dependent upon kidney function because TML availability is probably the rate-dependent step in carnitine biosynthesis, or at least TML clearance. [15], [28] Inclusion of eGFR in our multivariate models did however not substantially alter the TML effect; neither did inclusion of carnitine precursor γ-butyrobetaine or carnitine, suggesting that the observed association between TML and CAD progression is not confounded by neither kidney function nor metabolites of carnitine biosynthesis. Accordingly, carnitine metabolites did not predict CAD progression in a separate analysis.

While the adverse CVD-consequence of ADMA has been linked to NO inhibition, there is no indication that TML acts by a similar mechanism. It is known that FA supplementation increases the availability of SAM and it has recently been shown that FA supplementation induces aberrant DNA methylation in vitro [7] Increased SAM, induced by FA, may enhance methylation of histones, but data supporting such effects have not been published. Histone methylation can be either mono-, di- or trimethylated, all of which can alter gene expression. [6] Since di- and trimethylated histones are precursors for ADMA and TML respectively, one could speculate if levels of ADMA and TML reflect global histone hypermethylation.

While we have previously reported a possible detrimental effect of FA treatment in a sub-group of patients, [5] we were unable to prove an association between treatment with FA/vitamin B_12_ and plasma levels of either TML or ADMA. Thus, this observation does not substantiate a possible relationship between histone hypermethylation and plasma levels ADMA or TML.

Because QCA is a lumenogram, it provides no coronary plaque information, for which intravascular ultrasound imaging or optical coherence tomography would be appropriate. However, QCA is considered an accurate method of measuring progression or regression of coronary atherosclerosis over time [29] as well as being a reliable predictor of in-trial and post-trial clinical events [18], [19], [30] and in some respect possibly superior to intravascular ultrasound imaging. [31] Another limitation is the lack of data on global DNA or histone methylation status which makes us unable to conclude whether TML or ADMA reflect enhanced methylation during supplementation with FA. While FA has shown to induce DNA hypermethylation, [7] effect on histone methylation following treatment with FA has not been reported.

Our study is amongst the larger studies using QCA [18], [19], [32], [33]. By performing all QCA analyses twice by 2 technicians and averaging the results, the precision of data analysis was increased. DS was chosen as the appropriate variable, since it, in contrast to minimum lumen diameter, is a relative measurement, thus reducing any potential calibration errors between the baseline and follow-up angiograms. In addition the statistical methods applied allow us to reduce the number of data assumptions. Mixed effects modeling was applied to adjust for the intra-patient correlation between coronary artery lesions, and quantile regression was used to enable non-parametric model allowing for untransformed data without having to assume normality; both methods leading to greater statistical robustness. [24] LQMM also allowed us to model the entire distribution of the outcome variable presenting a more total and unselected view of the data and illuminating any possible tail effects. [34] The modest ICCs of 0.37 for TML and 0.54 for ADMA, may be related to strict metabolic regulation [35] and the influence of life-style factors such as diet and physical activity on plasma levels. Since risk estimates based single measurements tends to underestimate the true effect due to regression dilution one could suspect that the “true” relationships of ADMA and TML with DS are even stronger. [36].

In this prospective study of patients with established CAD both ADMA and TML was significantly and independently associated with angiographic progression of CAD in multivariate statistical models, although with a slight asymmetrical effect for TML and with ADMA showing the strongest relationship. However, moderate doses of FA and vitamin B_12_ did not alter the plasma levels of ADMA or TML except those in the lower 10^th^ percentile of ADMA. Since degradation of methylated proteins such as histones is the sole source of TML and ADMA, one could speculate whether ADMA and TML are markers of global histone methylation. Further investigation should aim at identifying (lifestyle) determinants of plasma TML, effect on cardiovascular end-points and whether TML levels modify the effect of B-vitamin treatment.
